# First Report of *Desmodium styracifolium* as a Novel Host for ‘*Candidatus Phytoplasma australasiaticum*’—Related Strains in China

**DOI:** 10.3390/microorganisms14030657

**Published:** 2026-03-14

**Authors:** Yafei Tang, Zhenggang Li, Mengdan Du, Guobing Lan, Lin Yu, Shanwen Ding, Zifu He, Xiaoman She

**Affiliations:** Guangdong Provincial Key Laboratory of High Technology for Plant Protection, Plant Protection Research Institute, Guangdong Academy of Agricultural Sciences, Guangzhou 510640, China

**Keywords:** phytoplasma, *Desmodium styracifolium*, molecular detection, 16SrII-A, new host

## Abstract

*Desmodium styracifolium* (Osb.) Merr., a member of the *Leguminosae* family, is an important medicinal plant widely used in traditional Chinese medicine. In September 2024, *D. styracifolium* plants exhibiting symptoms of little leaf and stunted growth were observed in a field of Zhanjiang, Guangdong province, China. Since the symptoms resembled those associated with phytoplasma infections, total DNA was extracted from the leaves of four symptomatic plants and one healthy plant for molecular identification. Universal primer pairs (P1/P7, R16mF2/mR1) for phytoplasma detection were used to amplify the 16S rDNA fragments (~1.8 kb and ~1.4 kb), while a specific primer pair secY-F/secY-R was employed to amplify a ~1.4 kb segment of the *secY* gene. Target fragments were successfully amplified from all symptomatic samples but not from the healthy control. These amplicons were cloned and sequenced. The obtained *16S rDNA* sequence of *D. styracifolium* little leaf phytoplasma (DsLFP-GDZJ) showed the highest identity (99.67–100%) with strains of ‘*Ca.* Phytoplasma australasiaticum’ (subgroup 16SrII-A and 16SrII-D). Phylogenetic analysis also indicated that DsLFP-GDZJ formed a small evolutionary branch with strains of ‘*Ca.* Phytoplasma australasiaticum’ (subgroup 16SrII-A and 16SrII-D). Virtual RFLP (restriction fragment length polymorphism) analysis of the 16S *rDNA* sequence demonstrated DsLFP-GDZJ belongs to the 16SrII-A subgroup (GenBank accession number L33765). The *secY* gene sequence of DsLFP-GDZJ also showed the highest similarity and the closest relationship with those of the 16SrII-A subgroup phytoplasma strains. These results showed that DsLFP-GDZJ is a strain of ‘*Ca.* Phytoplasma australasiaticum’ (16SrII-A subgroup). To our knowledge, this is the first report of ‘*Ca.* Phytoplasma australasiaticum’—related phytoplasma associated with *D. styracifolium* little leaf disease in China, thereby establishing *D. styracifolium* (Osb.) Merr. as a new host plant of phytoplasma.

## 1. Introduction

Phytoplasmas, which belong to the genus *Candidatus Phytoplasma* within the class Mollicutes, are significant obligate prokaryotic bacteria that lack a cell wall and cannot be cultured outside of their hosts. These organisms exhibit pleomorphic forms, with sizes ranging from 200 to 800 nm, and possess relatively small genomes varying from 680 to 1600 kb [1,2]. They primarily proliferate within plant phloem tissues and insect haemolymph [3,4]. Phytoplasmas are transmitted by insect vectors, mainly leafhoppers, planthoppers, and psyllids, as well as through dodder and grafting. Notably, recent studies have also identified that phytoplasma can be transmitted by seed [5,6,7,8,9,10]. Phytoplasmas have a wide host range and can infect thousands of plant species, including fruits, vegetables, ornamental plants, and shade and timber trees [11]. Infected plants exhibit a series of typical symptoms such as phyllody, virescence, witches’ broom, yellowing, little leaf, proliferation, necrosis, dieback, stunting, and bunchy top [8,9,12,13,14]. Phytoplasmas have constituted a class of highly damaging plant pathogens, posing a significant constraint on productivity and quality in global agriculture and forestry [1,15].

Symptom observation and molecular detection are widely utilized approaches for phytoplasma disease diagnosis. Recently, *16S* *rRNA* gene-based molecular analysis has been used to identify and classify phytoplasmas. If the full-length or nearly full-length 16S *rDNA* sequence (>1500 bp) of a strain sharing greater than 98.65% nucleotide identity with the reference strain is considered a member of the respective ‘*Ca.* Phytoplasma’ species. When the *16S rDNA* sequence alone is insufficient to differentiate ‘*Ca.* Phytoplasma’ species, additional conserved or housekeeping genes, such as *rp*, *tuf*, *secY*, and *secA*, are suggested to confirm or support the ‘*Ca*. Phytoplasma’ species identification. The threshold for these genes is set at 97.5% for *rp* and *tuf*, 95.7% for *secY*, and 95.0% for *secA*, enabling clear differentiation among them [2,16]. The classification into groups and subgroups is based on restriction fragment length polymorphism (RFLP) patterns of the F2nR2 fragment (about 1250 bp) of the 16S *rDNA*. To date, 49 named ‘*Ca*. Phytoplasma’ species have been identified [2,16,17].

*Desmodium styracifolium* (Osb.) Merr. is a member of the *Desmodium* genus within the *Leguminosae* family. In China, it is known as “Guang Jin qian cao” and is an important medicinal plant widely used in traditional Chinese medicine for treating many different human diseases [18,19]. To date, only two *Desmodium* plants have been reported to be infected by phytoplasma, *D. ovalifolium* and *D. triflorum*. *D. ovalifolium* witches’ broom phytoplasma was found in Hainan Province, and *D. triflorum* little leaf phytoplasma was found in Taiwan [20].

In this study, *D. styracifolium* plants showing typical little leaf symptoms were found in Zhanjiang city of Guangdong province, China. To identify the causative phytoplasma, molecular detection, phylogenetic, and virtual RFLP (restriction fragment length polymorphism) analyses were employed to classify phytoplasmas infecting these symptomatic plants. Our findings represent the first report of a phytoplasma associated with *D. styracifolium* little leaf disease in China. This study will contribute to a better understanding of the distribution and molecular diversity of phytoplasmas in China.

## 2. Materials and Methods

### 2.1. Field Investigation and Sample Collection

In September 2024, *D. styracifolium* plants exhibiting little leaf symptoms were found in Zhanjiang city of Guangdong province, China (21°15′8″ N, 110°6′11″ E). Disease incidence was investigated according to symptoms observed in the field. Leaf samples were randomly collected from four diseased plants and one healthy plant.

### 2.2. DNA Extraction and Molecular Identification

Total DNA was extracted from the collected five leaf samples using the Easypure Plant Genomic DNA Kit (Beijing TransGen Biotech Co., Beijing, China). PCR detection was performed with universal ribosomal primers P1/P7 [21,22] and R16mF2/R16mR1 [23] targeting the 16S-23S ribosomal gene, as well as the specific primers secY-F/secY-R (5′-GCGGAAGAAGCTATTAT-3′/5′-CGAATAACATAATATAATTGATTCC-3′) for the *secY* gene [24]. The amplification protocols are 94 °C for 10 min, 35 cycles each of 94 °C for 30 s, 50 °C for 30 s, and 72 °C for 2 min, and a final extension at 72 °C for 10 min. PCR products were analyzed by electrophoresis on 1% agarose gel, visualized under a UV transilluminator (Bio-Rad, Hercules, CA, USA). The amplified fragments were purified and ligated into the pMD-19T vector (Takara Bio Inc., Kusatsu, Japan). The ligation mixtures were transformed into *Escherichia coli* DH5α competent cells using the heat shock method. Positive clones were confirmed by bidirectional DNA sequencing by the Sanger method at Sangon Biotech Co. (Shanghai, China).

### 2.3. Sequence Analysis

Initial identification of ‘*Ca*. Phytoplasma’ species was performed by sequence comparison using the BLASTn algorithm in the GenBank database (www.ncbi.nlm.nih.gov, accessed on 20 May 2025). The homology analysis of *16S rRNA* and *secY* gene sequences was performed using the online MUSCLE tool (https://www.ebi.ac.uk/Tools/msa/muscle/, accessed on 27 May 2025). Phylogenetic trees were constructed using the neighbor-joining method implemented in MEGA 6.0 with 1000 bootstrap replicates. Two separate datasets retrieved from the GenBank database were analyzed: one comprising the *16S rRNA* gene sequences of DsLFP-GDZJ and 30 other phytoplasma strains (Table S1), and the other comprising the *secY* gene sequences of DsLFP-GDZJ and 19 other phytoplasma strains (Table S2). Virtual RFLP analysis of the F2nR2 fragment of the *16S rRNA* gene was conducted to define the 16Sr group and subgroup using the online interactive tool *i*PhyClassifier (https://plantpathology.ba.ars.usda.gov/cgi-bin/resource/iphyclassifier.cgi, accessed on 27 May 2025) [25].

## 3. Results

### 3.1. Field Surveys

**Figure 1:**
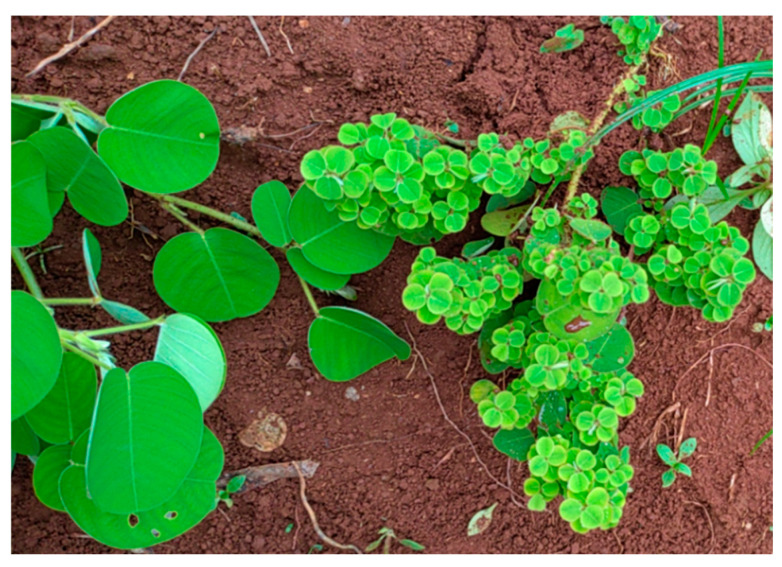
Little leaf symptom of the *D. styracifolium* plant. Left: healthy plant. Right: diseased plant.

In September 2024, *D. styracifolium* plants in the Mazhang district of Zhanjiang, Guangdong province, China, displayed typical little leaf symptoms, characterized by the emergence of abnormally small leaves (Figure 1). These symptoms strongly indicated a potential phytoplasma infection. About 300 plants were surveyed in the field, among which 10 exhibited symptoms, resulting in a disease incidence of 3.33%.

### 3.2. Molecular Detection

**Figure 2:**
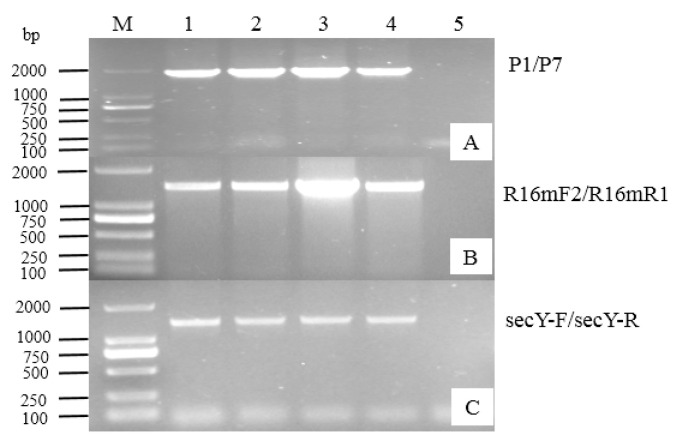
PCR detection of phytoplasma infection in *D. styracifolium* plants. (**A**): PCR amplification of *16S rDNA* with primer pair P1/P7 (1.8 kb products). (**B**): PCR amplification of *16S rDNA* with primer pair R16mF2/mR1 (1.4 kb products). (**C**): PCR amplification of the *secY* gene with primer pair secY-F/secY-R (1.4 kb products). M: 2000 bp DNA marker. Lane 1–4: diseased plants. Lane 5: healthy plants.

Four symptomatic and one healthy sample were detected by PCR using phytoplasma *16S rDNA* universal primer pairs, P1/P7 and R16mF2/mR1. The expected fragments of approximately 1.8 or 1.4 kb were obtained from four symptomatic samples, whereas no fragment was obtained from the healthy sample (Figure 2). The PCR result indicated that these *D. styracifolium* plants with little leaf were infected by phytoplasma. To further verify the presence of phytoplasma, we employed a specific primer pair, secY-F/secY-R, to amplify the *secY* gene. The PCR results also showed that the expected fragment of approximately 1.4 kb was obtained from all diseased samples, with no fragment in the healthy sample (Figure 2). These results conclusively confirmed the presence of phytoplasma in these diseased *D. styracifolium* plants. The identified phytoplasma strain was tentatively named DsLFP-GDZJ.

### 3.3. Sequence Analysis of 16S rDNA

The target fragment amplified using primer pair P1/P7 was gel-purified, cloned, and sequenced. The fragment amplified from the total DNA of *D. styracifolium* symptomatic samples in Guangdong was 1806 bp in length. The nucleotide sequence was submitted to the National Center for Biotechnology Information (NCBI) to get the GenBank accession number PV546888. Sequence analysis revealed that the fragment contains the *16S rDNA* (1–1525 nt), the 16S-23S rDNA intergenic spacer region (1526–1751 nt), which includes the *rDNA-Ile* gene (1604–1680 nt), and a partial segment of the 23S *rDNA* (1752–1806 nt).

BLAST analysis revealed that the *16S rDNA* sequence of DsLFP-GDZJ shared the highest identity with those of phytoplasma strains belonging to the peanut witches’ broom group (16SrII group). Further, MUSCLE analysis results (Table 1) showed that the *16S rDNA* sequence of DsLFP-GDZJ shared the highest identity (99.67–100%) with those of ‘*Ca.* Phytoplasma australasiaticum’ (subgroup 16SrII-A and 16SrII-D) strains. Among them, DsLFP-GDZJ shared 100% identity with 11 phytoplasma strains associated with adzuki bean witches’ broom (PQ658233), eggplant phyllody (MH667642), *Cleome rutidosperma* witches’ broom (OP875099), soybean witches’ broom (MW680828), *Crotalaria* witches’ broom (EU650181), *Chrysanthemum* virescence (AB247462), sweet potato little leaf (AJ289193), *Desmodium ovalifolium* witches’ broom (GU113152 and MK956144), *Desmodium triflorum* little leaf (MT452308), and pear decline (EF193157). The identity with other strains (subgroup 16SrII-B, 16SrII-C, and 16SrII-L) ranged from 98.00% to 98.56%. In contrast, the similarity with strains from the ash yellows group (16SrVII group), elm yellows group (16SrV group), and aster yellows phytoplasma group (16SrI group) was much lower (89.25–91.12%). These results indicate that DsLFP-GDZJ is a strain related to ‘*Ca.* Phytoplasma australasiaticum’.

**Figure 3:**
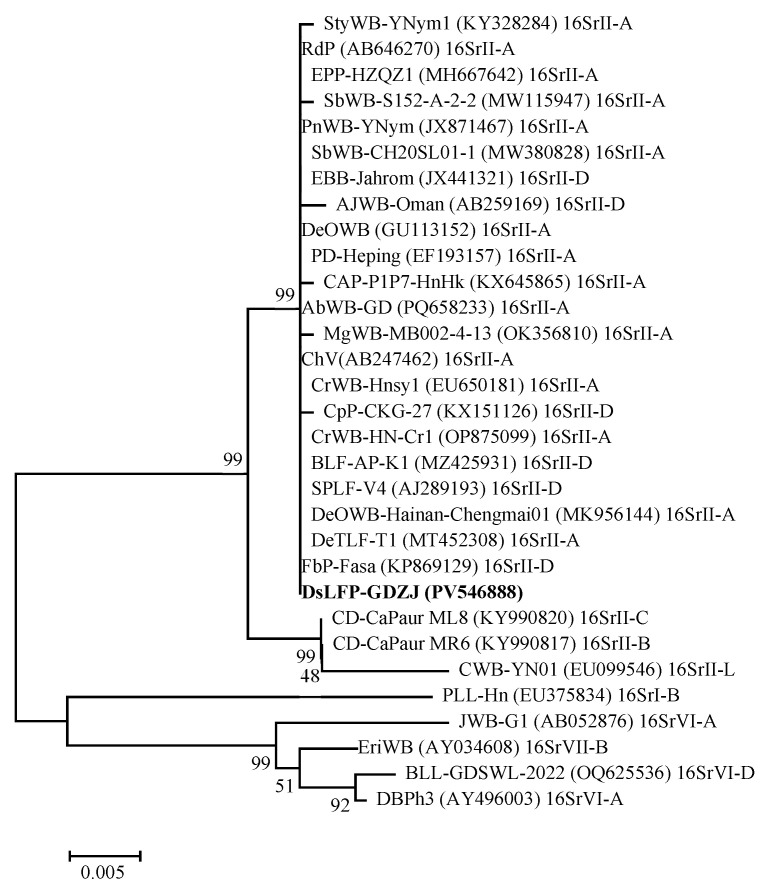
Phylogenetic tree depicting the relationships of the *16S rDNA* sequences of DsLFP-GDZJ and 30 other phytoplasma strains retrieved from the GenBank database. The tree was constructed using the neighbor-joining method implemented in MEGA 6.0. The bootstrap consensus tree was inferred from 1000 iterations. DsLFP-GDZJ identified in this study is marked in bold.

The phylogenetic analysis (Figure 3) of *16S rDNA* revealed that DsLFP-GDZJ was clustered within a major branch alongside 25 related phytoplasma strains belonging to the 16SrII group, indicating a close phylogenetic relationship. In contrast, it showed a distant relationship with strains from groups 16SrI, 16SrVI, and 16SrVII. Further analysis revealed that DsLFP-GDZJ formed a tight subclade with strains from 16SrII-A and 16SrII-D subgroups, demonstrating the closest genetic affinity.

**Figure 4:**
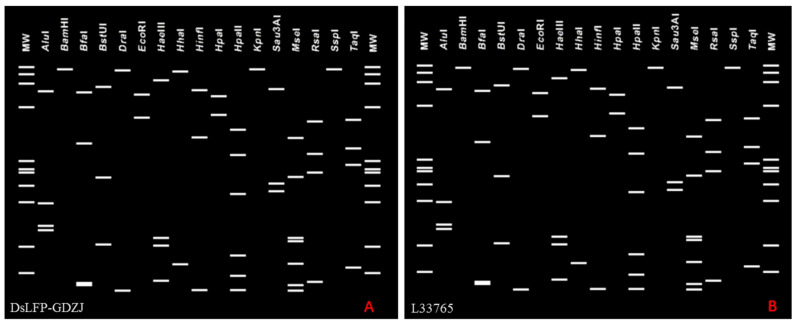
Virtual RFLP patterns of DsLFP-GDZJ and the reference strain of the 16SrII-A subgroup (L33765) based on the *16S rDNA* fragment. (**A**) DsLFP-GDZJ; (**B**) reference strain of the 16SrII-A subgroup (L33765).

The online phytoplasma classification tool, *i*PhyClassifier, was used to conduct the virtual RFLP analysis of the F2nR2 fragment of the DsLFP-GDZJ *16S rRNA* gene. The results (Figure 4) showed that DsLFP-GDZJ exhibited an identical restriction pattern to the reference strain of subgroup 16SrII-A (L33765), with a similarity coefficient of 1.00, which further demonstrates that DsLFP-GDZJ belongs to the 16SrII-A subgroup.

### 3.4. Sequence Analysis of the secY Gene

The target fragment amplified using primer pair secY-F/secY-R was gel-purified, cloned, and sequenced. The amplified fragment from diseased *D. styracifolium* samples in Guangdong was 1425 bp in length, and contained the full length of the *secY* gene (74–1336 nt). The nucleotide sequence was submitted to NCBI to get the GenBank accession number PV553464.

**Figure 5:**
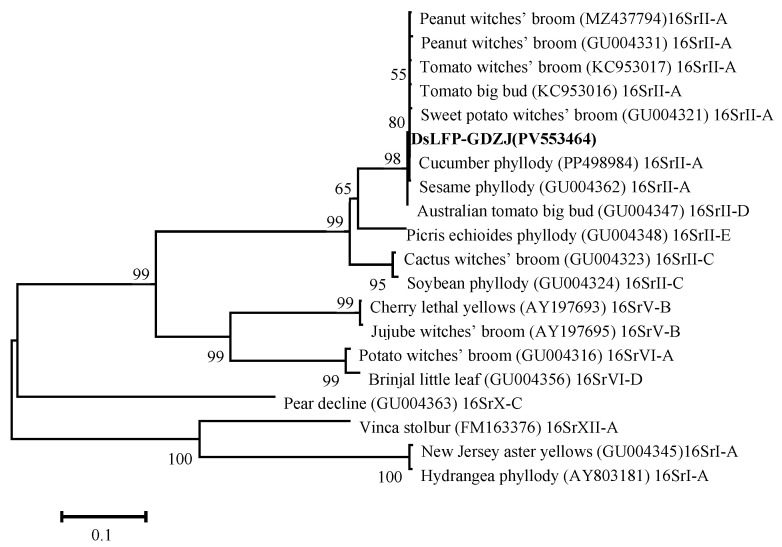
Phylogenetic tree showing relationships of the *secY* sequences of DsLFP-GDZJ to other 19 phytoplasma strains retrieved from the GenBank database. The tree was reconstructed using the neighbor-joining method implemented in MEGA 6.0. The bootstrap consensus tree was inferred from 1000 iterations. DsLFP-GDZJ identified in this study is marked in bold.

The BLAST analysis revealed that the *secY* sequence of DsLFP-GDZJ shared 100% identity with several phytoplasma strains belonging to the 16SrII group, including four strains: NCHU2014, NCHU2022, WF_GM2021, and SPWB of *Ca.* Phytoplasma australasiaticum (CP040925, CP097312, CP133702, CP171825), and other strains associated with *Vigna angularis* witches’ broom (PQ619118), *Perilla frutescens* witches’ broom (MW310228), cowpea virescence (KC953013), *Gendarussa vulgaris* witches’ broom (MN543069), cauliflower phyllody (KC953012), long bean phyllody (AB703251), cucumber phyllody (PP498984), black gram witches’ broom (AB703249), *Crotalaria* witches’ broom (JF834194), and shaggy button witches’ broom (AB703252). Furthermore, the phylogenetic tree constructed based on *secY* gene sequences of DsLFP-GDZJ and 19 other phytoplasmas showed that the DsLFP-GDZJ strain was clustered with seven strains belonging to subgroup 16SrII-A and one strain belonging to subgroup 16SrII-D (Figure 5). These findings collectively suggest that the DsLFP-GDZJ strain is a member of the 16SrII-A subgroup of phytoplasmas.

## 4. Discussion

Phytoplasma diseases are one of the most devastating plant diseases worldwide. Phytoplasmas can infect over 1000 plant species, including critical food crops, vegetables, and fruit trees, often causing severe yield losses and substantial economic damage [11]. In China, over 100 different plant species have been reported to be infected by diverse phytoplasmas, resulting in significant economic losses to agricultural and forestry production [26].

In 2024, *D. styracifolium* plants exhibiting little leaf symptoms were first observed at a field in China. PCR testing confirmed that the samples were infected with a phytoplasma, and the strain was tentatively designated as *D. styracifolium* little leaf phytoplasma (DsLFP-GDZJ). The *16S rDNA* sequence of DsLFP-GDZJ showed 99.67% to 100% similarity with strains of ‘*Ca.* Phytoplasma australasiaticum’ from 16SrII-A and 16SrII-D subgroups. The phylogenetic analysis of *16S rDNA* also revealed that DsLFP-GDZJ is clustered into 16SrII-A and 16SrII-D subgroups. According to the *i*PhyClassifier analysis results, DsLFP-GDZJ belonged to the 16SrII-A subgroup. According to current international phytoplasma classification standards [16], DsLFP-GDZJ is a new strain of ‘*Ca.* Phytoplasma australasiaticum’ (belonged to the 16SrII-A group). The *secY* gene sequence analysis also revealed that DsLFP-GDZJ shared the highest similarity and closest phylogenetic relationship with strains of the 16SrII-A subgroup. This study represents the first report of a phytoplasma disease on *D. styracifolium*, specifically caused by a strain of ‘*Ca.* Phytoplasma australasiaticum’.

In China, phytoplasmas display high genetic diversity and a wide geographical distribution, with 12 distinct groups identified, namely 16SrI, 16SrII, 16SrV, 16SrVII, the X-disease group (16SrIII), clover proliferation group (16SrVI), loofah witches’ broom group (16SrVIII), apple proliferation group (16SrX), rice yellow dwarf group (16SrXI), stolbur group (16SrXII), bermudagrass white leaf group (16SrXIV), and Malaysian periwinkle virescence group (16SrXXXII) [20,26]. Guangdong province is located in the tropical and subtropical regions of southern China and is renowned for its abundant biological resources and genetic diversity. However, Guangdong also suffers significantly from phytoplasma-associated diseases. To date, at least four groups (16SrI, 16SrII, 16SrV, and 16SrVI) of phytoplasmas have been reported to be distributed in Guangdong. Specifically, 16SrI phytoplasma was associated with rice orange leaf, mulberry yellow dwarf, gum tree yellowing, and witches’ broom [27,28,29]. 16SrV phytoplasma was associated with jujube witches’ broom [30]; 16SrVI phytoplasma with *Breynianivosa* little leaf [31]; and 16SrII phytoplasmas with eggplant phyllody, peanut witches’ broom, cowpea phyllody, tomato big bud, and cucumber phyllody [31,32,33,34,35,36]. Thus, the 16SrII phytoplasmas may represent the dominant group affecting *Solanaceae*, *Leguminosae*, and *Cucurbitaceae* in Guangdong, China. This study reveals that *D. styracifolium* (Osb.) Merr. serves as a new host plant for phytoplasmas of the 16SrII-A subgroup.

This study contributes to understanding the distribution, host range, and molecular diversity of phytoplasmas present in China. Phytoplasmas are mainly transmitted to plants by insects such as leafhoppers, planthoppers, psyllids, etc. To prevent serious crop losses caused by phytoplasmas, disrupting the transmission pathways is the most effective strategy. Therefore, further studies should be performed to investigate various transmission routes of phytoplasmas and plan, develop, and implement effective vector management and disease control strategies.

## 5. Conclusions

In 2024, a new disease was discovered in *D. styracifolium* plants in the fields of Zhanjiang city, Guangdong province, characterized by typical phytoplasma-induced symptoms such as little leaf. The causative phytoplasma, designated as DsLFP-GDZJ, was detected and identified using molecular biology methods. According to the results of sequence similarity analysis and phylogenetic analysis of *16S rRNA* and *secY* genes, and virtual RFLP analysis of the *16S rRNA* gene, DsLFP-GDZJ is a strain of ‘*Ca.* Phytoplasma australasiaticum’, and belongs to the 16SrII-A subgroup. To our knowledge, this is the first report of ‘*Ca.* Phytoplasma australasiaticum’ (16SrII-A group) associated with *D. styracifolium* little leaf disease in China, thereby establishing *D. styracifolium* (Osb.) Merr. as a new host plant of phytoplasma.

## Figures and Tables

**Table 1:** Identities of *16S rDNA* sequences among DsLFP-GDZJ and related phytoplasmas.

Plant Disease Name	Strain	16S rDNA Group/Subgroup	Origin	GenBank Accession No.	Identity (%)
Adzuki bean witches’ broom	AbWB-GD	16SrII-A	China	PQ658233	100.00
Eggplant phyllody	EPP-HZQZ1	16SrII-A	China	MH667642	100.00
Cleome rutidosperma witches’ broom	CrWB-HN-Cr1	16SrII-A	China	OP875099	100.00
Soybean Witches’ Broom	SbWB-CH20SL01-1	16SrII-A	China	MW680828	100.00
*Crotalaria* witches’ broom	CrWB-Hnsy1	16SrII-A	China	EU650181	100.00
*Chrysanthemum* virescence	ChV	16SrII-A	Okinawa	AB247462	100.00
Sweet potato little leaf	SPLF-V4	16SrII-A	Australia	AJ289193	100.00
*Desmodium ovalifolium* witches’ broom	DeOWB	16SrII-A	China	GU113152	100.00
*Desmodium ovalifolium* witches’ broom	DeOWB-Hainan-Chengmai01	16SrII-A	China	MK956144	100.00
*Desmodium triflorum* little leaf	DeTLF-T1	16SrII-A	China	MT452308	100.00
Pear decline	PD-Heping	16SrII-A	China	EF193157	100.00
*Corchorus aestuans* phyllody	CAP-P1P7-HnHk	16SrII-A	China	KX645865	99.93
Soybean witches’ broom	SbWB-S152-A-2-2	16SrII-A	China	MW115947	99.93
Mungbean Witches’ Broom	MgWB-MB002-4	16SrII-A	China	OK356810	99.93
Peanut witches’ broom	PnWB-YNym	16SrII-A	China	JX871467	99.93
*Stylosanthes guianensis* witches’ broom	StyWB-YNym1	16SrII-A	China	KY328284	99.87
Brinjal little leaf	BLF-AP-K1	16SrII-D	India	MZ425931	99.87
Chickpea phyllody	CpP-CKG-27	16SrII-D	India	KX151126	99.80
Radish phyllody	RdP	16SrII-A	Myanmar	AB646270	99.80
Faba bean phyllody	FbP-Fasa	16SrII-D	Iran	KP869129	99.74
Eggplant big bud	EBB-Jahrom	16SrII-D	Iran	JX441321	99.67
Arabian Jasmine witches’ broom	AJWB-Oman	16SrII-D	Oman	AB259169	99.67
Citrus decline	CD-CaPaur_ML8	16SrII-C	Iran	KY990820	98.56
Citrus decline	CD-CaPaur_MR6	16SrII-B	Iran	KY990817	98.56
Cactus witches’ broom	CWB-YN01	16SrII-L	China	EU099546	98.00
Erigeron witches’ broom	EriWB	16SrVII-B	Brazil	AY034608	91.12
Dry bean phyllody	DBPh3	16SrVI-A	USA	AY496003	90.90
*Breynianivosa* little leaf	BLL-GDSWL-2022	16SrVI-D	China	OQ625536	90.65
*Periwinkle little* leaf	PLL-Hn	16SrI-B	China	EU375834	89.25

## Data Availability

The original contributions presented in this study are included in the article/Supplementary Material. Further inquiries can be directed to the corresponding authors.

## Supplementary Materials

The following supporting information can be downloaded at: https://www.mdpi.com/article/10.3390/microorganisms14030657/s1. Table S1: *16S rRNA* sequences of 30 other phytoplasma strains for phylogenetic analysis; Table S2: *secY* gene sequences of 19 other phytoplasma strains for phylogenetic analysis.
