# Supplementary material for: First Report of Desmodium styracifolium as a Novel Host for ‘Candidatus Phytoplasma australasiaticum’—Related Strains in China
Source: Microorganisms. 2026 Mar 14;14(3):657. doi: 10.3390/microorganisms14030657 (PMC13029475; doi:10.3390/microorganisms14030657)
Supplement: Supplementary file 1 [file microorganisms-14-00657-s001.zip › Supplementary Table S1.pdf]

Supplementary Table S1 *16S rRNA* sequences of 30 other phytoplasma strains for phylogenetic analysis

Plant disease name	strain	16S rDNA group/subgroup	ORIGIN	GenBank accession No.
adzuki bean witches'- broom	AbWB-GD	16SrII-A	China	PQ658233
eggplant phyllody	EPP-HZQZ1	16SrII-A	China	MH667642
Cleome rutidospermawitches'-broom	CrWB-HN-Cr1	16SrII-A	China	OP875099
Soybean Witches'-Broom	SbWB-CH20SL01-1	16SrII-A	China	MW680828
Crotalaria witches'-broom	CrWB-Hnsy1	16SrII-A	China	EU650181
*Chrysanthemum*virescence	ChV	16SrII-A	Okinawa	AB247462
Sweet potato little leaf	SPLF-V4	16SrII-A	Australia	AJ289193
*Desmodiumovalifolium*witches'-broom	DeOWB	16SrII-A	China	GU113152
*Desmodiumovalifolium*witches'-broom	DeOWB-Hainan-Chengmai01	16SrII-A	China	MK956144
Desmodiumtriflorum little leaf	DeTLF-T1	16SrII-A	China	MT452308
Pear decline	PD-Heping	16SrII-A	China	EF193157
Corchorus aestuans phyllody	CAP-P1P7-HnHk	16SrII-A	China	KX645865
Soybean witches'-broom	SbWB-S152-A-2-2	16SrII-A	China	MW115947
Mungbean Witches' Broom	MgWB-MB002-4	16SrII-A	China	OK356810
Peanut witches'-broom	PnWB-YNym	16SrII-A	China	JX871467
*Stylosanthesguianensis*witches'-broom	StyWB-YNym1	16SrII-A	China	KY328284
Brinjal little leaf	BLF-AP-K1	16SrII-D	India	MZ425931
Chickpea phyllody	CpP-CKG-27	16SrII-D	India	KX151126
Radish phyllody	RdP	16SrII-A	Myanmar	AB646270
Faba bean phyllody	FbP-Fasa	16SrII-D	Iran	KP869129
Eggplant big bud	EBB-Jahrom	16SrII-D	Iran	JX441321
Arabian Jasmine witches'-broom	AJWB-Oman	16SrII-D	Oman	AB259169
Citrus decline	CD-CaPaur_ML8	16SrII-C	Iran	KY990820
Citrus decline	CD-CaPaur_MR6	16SrII-B	Iran	KY990817
Cactus witches'-broom	CWB-YN01	16SrII-L	China	EU099546
Erigeron witches'-broom	EriWB	16SrVII-B	Brazil	AY034608
Dry bean phyllody	DBPh3	16SrVI-A	USA	AY496003
Jujube witches'-broom	JWB-G1	16SrVI-A	Japan	AB052876
Breynianivosa little leaf	BLL-GDSWL-2022	16SrVI-D	China	OQ625536
Periwinkle little leaf	PLL-Hn	16SrI-B	China	EU375834
