# Supplementary material for: First Report of Desmodium styracifolium as a Novel Host for ‘Candidatus Phytoplasma australasiaticum’—Related Strains in China
Source: Microorganisms. 2026 Mar 14;14(3):657. doi: 10.3390/microorganisms14030657 (PMC13029475; doi:10.3390/microorganisms14030657)
Supplement: Supplementary file 1 [file microorganisms-14-00657-s001.zip › Supplementary Table S2.pdf]

Supplementary Table S2 *secY gene* sequences of 19 other phytoplasma strains for phylogenetic analysis

Plant disease name	strain	16S rDNA group/subgroup	ORIGIN	GenBank accession No.
Peanut witches'-broom	PnWB-GDSX-2020	16SrII-A	China	MZ437794
Peanut witches'-broom	PnWB-GDSX-2020	16SrII-A	China	GU004331
Tomato witches'-broom	TWB-YNym	16SrII-A	China	KC953017
Tomato big bud	TBB- YNym	16SrII-A	China	KC953016
Sweet potato witches'-broom	SPWB	16SrII-A	China	GU004321
Cucumber phyllody	CuPh-MM	16SrII-A	China	PP498984
Sesame phyllody	SEPN	16SrII-A	Thailand	GU004362
Australian tomato big bud	TBB	16SrII-D	Australia	GU004347
Picris echioides phyllody	PEP	16SrII-E	Italy	GU004348
Cactus witches'-broom	CWB-YN16	16SrII-C	China	GU004323
Soybean phyllody	SOYP	16SrII-C	Thailand	GU004324
Cherry lethal yellows	CLY-5	16SrV-B	China	AY197693
Jujube witches'-broom	JWB	16SrV-B	China	AY197695
Potato witches'-broom	PWB	16SrVI-A	Canada	GU004316
Brinjal little leaf	BLL	16SrVI-D	India	GU004356
Pear decline	PD1	16SrX-C	Italy	GU004363
Vinca stolbur	VS-P0	16SrXII-A	France	FM163376
New Jersey aster yellows	NJAY	16SrI-A	USA	GU004345
Hydrangea phyllody	HYDP	16SrI-A	Belgium	AY803181
